# Prediction of Ovarian Follicular Dominance by MRI Phenotyping of Hormonally Induced Vascular Remodeling

**DOI:** 10.3389/fmed.2021.711810

**Published:** 2021-08-20

**Authors:** Liat Fellus-Alyagor, Inbal E. Biton, Hagit Dafni, Filip Bochner, Ron Rotkopf, Nava Dekel, Michal Neeman

**Affiliations:** ^1^Department of Biological Regulation, Weizmann Institute of Science, Rehovot, Israel; ^2^Department of Veterinary Resources, Weizmann Institute of Science, Rehovot, Israel; ^3^Department of Life Science Core Facilities, Weizmann Institute of Science, Rehovot, Israel

**Keywords:** MRI, ovary, dominant follicle, corpus luteum, angiogenesis, permeability, blood volume, bi-modal imaging

## Abstract

In the mammalian female, only a small subset of ovarian follicles, known as the dominant follicles (DFs), are selected for ovulation in each reproductive cycle, while the majority of the follicles and their resident oocytes are destined for elimination. This study aimed at characterizing early changes in blood vessel properties upon the establishment of dominance in the mouse ovary and application of this vascular phenotype for prediction of the follicles destined to ovulate. Sexually immature mice, hormonally treated for induction of ovulation, were imaged at three different stages by dynamic contrast-enhanced (DCE) MRI: prior to hormonal administration, at the time of DF selection, and upon formation of the corpus luteum (CL). Macromolecular biotin-bovine serum albumin conjugated with gadolinium-diethylenetriaminepentaacetic acid (b-BSA-GdDTPA) was intravenously injected, and the dynamics of its extravasation from permeable vessels as well as its accumulation in the antral cavity of the ovarian follicles was followed by consecutive T_1_-weighted MRI. Permeability surface area product (permeability) and fractional blood volume (blood volume) were calculated from b-BSA-GdDTPA accumulation. We found that the neo-vasculature during the time of DF selection was characterized by low blood volume and low permeability values as compared to unstimulated animals. Interestingly, while the vasculature of the CL showed higher blood volume compared to the DF, it exhibited a similar permeability. Taking advantage of immobilized ovarian imaging, we combined DCE-MRI and intravital light microscopy, to reveal the vascular properties of follicles destined for dominance from the non-ovulating subordinate follicles (SFs). Immediately after their selection, permeability of the vasculature of DF was attenuated compared to SF while the blood volume remained similar. Furthermore, DFs were characterized by delayed contrast enhancement in the avascular follicular antrum, reflecting interstitial convection, whereas SFs were not. In this study, we showed that although DF selection is accompanied by blood vessel growth, the new vasculature remained relatively impermeable compared to the vasculature in control animal and compared to SF. Additionally, DFs show late signal enhancement in their antrum. These two properties may aid in clinical prediction of follicular dominance at an early stage of development and help in their diagnosis for possible treatment of infertility.

## Introduction

The oocytes, which are the female gametes, reside in the ovary while encapsulated by multi-cellular structures comprising together the ovarian follicles. In a sexually mature female, at any given time point, the ovary is populated by multiple follicles at different developmental stages (1–3). In the early primordial follicle, the oocyte is surrounded by a single layer of flattened granulosa cells. The development of primordial follicle into primary and secondary follicles at early folliculogenesis is characterized by proliferation of granulosa cells alongside the formation of an additional layer of theca cells, at the outer part of the follicle. Further development of the follicles from the preantral to the antral stages, is associated with the formation of a plasma exudate-filled cavity, known as the follicle antrum.

The majority of the female gametes, at all developmental stages, degenerate through a process known as atresia, while a small fraction continues their growth. During sexual maturation, the so far hormonally independent folliculogenic process acquires dependency on the cyclic secretion of the pituitary-derived gonadotropins. The gonadotropin follicle-stimulating hormone (FSH) induces growth of a subset of antral follicles up to the stage of the Graafian, also known as preovulatory, follicles. This event consists of upregulation of the receptors for another pituitary gonadotropin, luteinizing hormone (LH), as well as the production of estrogen, inhibin, and activin. The mid-cycle peak of LH released from the pituitary, known as the LH surge, will affect only these follicles expressing the LH receptor (LHR) to undergo the ovulatory changes. After follicle rupture and the subsequent release of the oocyte, collectively known as ovulation, the ovulating follicle will go through further differentiation to become the corpus luteum (CL) and will secrete progesterone, which supports the uterine endometrium preparation required for the establishment of pregnancy (2, 4). At each reproductive cycle, two populations of antral follicles develop, the follicles destined to ovulate, known as the dominant follicles (DFs) and the subordinate follicles (SFs), that will not ovulate and will undergo atresia. In the mouse, selection of DFs occurs 36 h after the exposure to FSH. At this stage, a sub-population of follicles, which expresses LHR, and thus is sensitive to this gonadotropin, had emerged (5).

Angiogenesis, defined as the sprouting of new blood vessels from pre-existing ones, plays a fundamental role in ovarian physiology and is essential for folliculogenesis, DF selection, and ovulation [reviewed in (6–9)]. The highly intensive dynamics of the angiogenic events that accompanies folliculogenesis is governed by vascular endothelial growth factor (VEGF) (10–15). Angiogenesis induced by VEGF is often characterized by increased vascular permeability, which allows vessel growth, followed by their re-stabilization upon pericyte recruitment. The rate of vessel permeability and their following re-stabilization vary between vessels, tissues, and physiological processes (16, 17).

During their development, follicles comprise a peripheral vascular network that is restricted to the theca cell layer. After ovulation, formation of the CL involves a massive angiogenic process, in which blood vessels penetrate into the so-far avascular inner parts of the follicle (18–22).

A leading hypothesis regarding the mechanism of DF selection and subsequent ovulation/CL formation suggests that DF advantage over the SF is facilitated by the development of a richer peripheral vasculature that amplifies the local exposure to gonadotropins. Along this line, increased vascularization of individual follicles in rhesus monkeys that resulted in preferential accumulation of gonadotropins has been demonstrated (23). Moreover, *in vivo* studies in cattle showed that maintenance of follicular vasculature and appropriate blood supply to follicles are essential for establishment of follicular dominance (24). In mares, differential blood flow and blood flow velocity are early parameters for identification of DF (25). One of the few studies that used rodents showed that larger follicles exhibit wider capillaries and higher capillary blood perfusion, supporting this hypothesis (26).

Most of the *in vivo* imaging studies on the ovarian vasculature were performed by color Doppler ultrasonography. Parameters, such as blood flow velocity in arterial and venous vessels, were characterized extensively in the ovaries of large animals such as cows (27, 28), ewes (29–31) and mares (25), and found to be associated with DF selection and CL formation; ultrasound imaging of the vasculature is also very common in women. However, the resolution provided by ultrasound methods in rodents is not sufficient for the visualization of small follicles. Moreover, small vessels are not properly represented by this method and the extracted parameters are limited mainly to blood flow-related ones.

Magnetic resonance imaging (MRI) offers higher resolution and provides multiple functional vessel parameters. The diffusion rate of fluids crossing from the vascularized theca layer to the antrum measured by MRI in rat follicles was found to decrease in preovulatory follicles (32). Changes in the perfusion of the ovary during the ovulatory response were also studied through pulsed arterial spin labeling MRI (33, 34). This method was also applicable for arterial blood velocity measurements. Vessel permeability and fractional blood volume were studied by dynamic contrast enhanced (DCE) MRI, using high-molecular-weight b-BSA-GdDTPA, in ovarian grafts for evaluation of the success of graft implantation and revascularization in the transplanted tissue (35–39). However, no such studies reported the dynamics of the angiogenic process that accompanies DF selection. Generating such information may assist in improving outcome for infertility treatments, particularly for patients that respond poorly to the hormonal treatment.

In this study, we investigated the dynamic changes in tissue concentration of b-BSA-GdDTPA during the angiogenic processes that accompany major events in ovarian physiology. We additionally compared blood volume and permeability of DF and SF shortly after dominance establishment, using multi-modality imaging acquired through an ovarian imaging window that allowed integration of high-resolution MRI with intravital microscopy (40, 41).

## Materials and Methods

### Animals

All experiments were carried out according to Israel regulations on animal experimentation and Weizmann Institute guidelines. All experimental protocols were reviewed and approved by Weizmann Institutional Animal Care and Use Committee (IACUC). Sexually immature C57BL/6J female mice (22–28 days old) were used for all experiments.

### Induction of Ovulation

Mice (C57BL/6J; Invigo, Jerusalem, Israel) were subcutaneously injected with 5 IU of the FSH analog, pregnant mare's serum gonadotropin (PMSG, National Hormone & Peptide Program, Harbor-UCLA Medical Center, California, U.S.A.; or Syncopart PMSG, Ceva, France) dissolved in 100 μl of Dulbecco's phosphate buffered saline (PBS). A dose of 5 IU of the LH analog, human chorionic gonadotropin (hCG, Sigma Aldrich, Rehovot, Israel) dissolved in 100 μl of PBS, was intraperitoneally administered to the animals 48 h after PMSG. Ovulation occurred ~12 h later.

### MRI of Ovaries Before and During DF Selection Process

Mice were studied at the sexually immature stage (non-injected, *n* = 6) and during DF selection, at 36–48 h after injection of PMSG (*n* = 5). Anesthesia was achieved by Isoflurane (5% for induction, 1–2% for maintenance) mixed with oxygen (1 L/min), delivered through a nasal mask. Respiration rate was monitored during MRI and kept throughout the experimental period at 60–80 breaths per minute. Body temperature was maintained at 37°C throughout the experiment using a circulating water blanket. Once anesthetized, a silicone catheter was inserted into the tail vein and the mice were placed at a head-supine position in a 9.4-T BioSpec Magnet 94/20 USR system (Bruker, Karlsruhe, Germany), equipped with gradient coil system capable of producing pulse gradient of up to 40 gauss/cm in each of the three dimensions. A linear volume coil was used for excitation and a 2-cm surface coil for detection (Bruker, Karlsruhe Germany). For the calculation of pre-contrast R1, a set of T_1_-weighted 3D-gradient echo (MDEFT) images (TR: 10 ms, TE =3.25 ms, two averages, matrix: 256 × 256 × 64, FOV: 30 × 30 × 32 mm, voxel size: 117 × 117 × 500 μm, scan time: 133 s) were acquired with varying flip angles (FA; 5°, 15°, 30°, 50°, and 70°). Then, b-BSA-GdDTPA [approximate MW is 82 kDa, final concentration in the blood = 100 mg/ml, ~0.8 g per kg; Symo Chem, Germany (42)] was injected intravenously through the pre-placed tail vein catheter (bolus injection over 1 min) and dynamic contrast enhancement was acquired using 3D T_1_-weighted images with a FA of 15° for a period of 35 min post injection (all other parameters are as stated above). The fluorescent tag carboxy-X-rhodamine (ROX, Molecular Probes, Oregon, USA) conjugated to bovine serum Albumin (BSA-ROX) was injected to the tail vein upon imaging completion. Mice were sacrificed 2 min later, before noticeable extravasation of BSA-ROX from the vessels, and their ovaries were collected for immunohistochemistry.

### MRI of Ovaries During DF Selection and After Ovulation

Mice were imaged by MRI during DF selection (36–48 h after PMSG injection, *n* = 8 mice, 12 ovaries). Mice were allowed to recover from anesthesia upon completion of imaging. Subsequently, ovulation was induced by hCG, and the same mice were anesthetized and imaged again 4–12 h after ovulation, at the time of corpora lutea (CL) formation. MRI was performed as mentioned above.

### Combined MRI and Microscopy Through Ovarian Imaging Window

**Figure 1:**
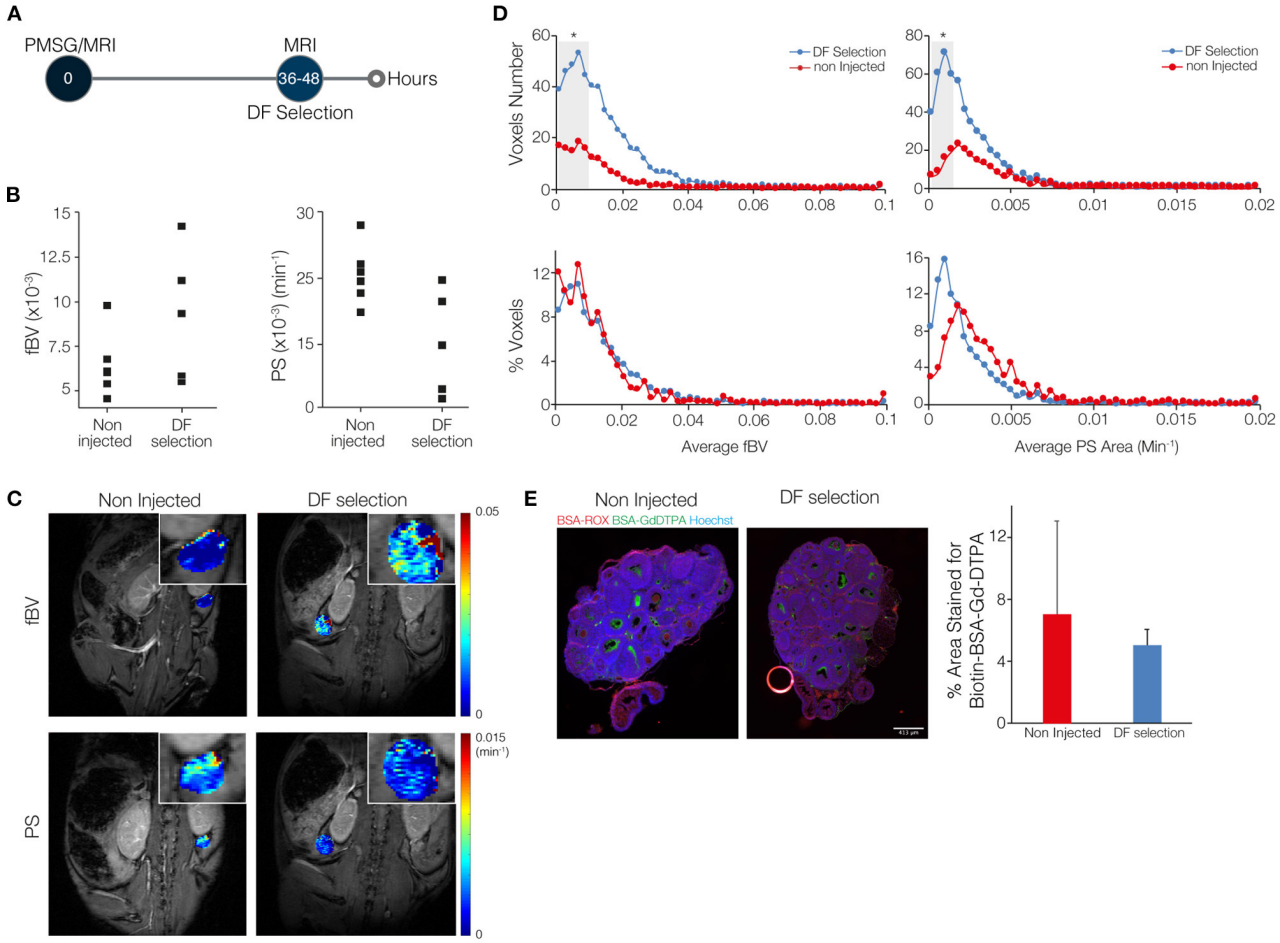
The angiogenic process that accompanies dominant follicle (DF) selection is characterized by a low blood volume and low permeability. **(A)** Mice were imaged by DCE-MRI 36–48 h after injection of PMSG (*n* = 5), soon after DF selection, and were compared to sexually immature non-injected control mice, with no DF in their ovaries (*n* = 6). **(B)** Fractional blood volume (fBV) and permeability surface area product (PS) averaged over the entire ovary voxels. **(C)** Maps of fBV and PS showing the value for each voxel in the ovary region of interest. Enlarged ovary region is shown in the inset. **(D)** Histogram analysis of the total number and percent of voxels in each value range of fBV and PS. Ranges with significant increase in voxels with low values of fBV and low values of PS are indicated in gray background (* represents *p*-value <0.05). **(E)** Fluorescent staining for the MRI contrast agent, b-BSA-GdDTPA (intra and extravascular at 35 min after injection; visualized using fluorescent streptavidin) and for BSA-ROX (confined mainly to the blood vessels at 2 min after injection). Red circle is an air bubble. Percent area covered by the b-BSA-GdDTPA staining was calculated.

High-resolution MRI was performed after mounting an MRI-compatible ovarian imaging window (40, 43) on sexually immature mice (*n* = 5). The ovary was carefully pulled out through a small incision in the peritoneum and placed inside the window by attaching the ovarian fat pad to the window with minimal amount of surgical glue (Vetbond, 3M, Maplewood, Minnesota, USA). After PMSG injection, at the time of DF selection, mice were anesthetized by a mix of Domitor (Medetomidine, 1 mg/kg, Vetoquinol, Lure Cedex, France) and Ketamine (75 mg/kg, Vetoquinol, Lure Cedex, France). Ovaries were imaged in the 9.4-T BioSpec MRI with a 10-mm 1H receive-only planar loop surface coil used in combination with a local preamplifier (Bruker, Germany) and 1H linear resonator for excitation. DCE-MRI was performed using T_1_-weighted 3D-gradient eco (FLASH) images (TR: 10 ms, TE: 3.25 ms, two averages, matrix: 512 × 512 × 48, FOV: 20 × 20 × 16 mm, pixel size: 39 × 39 × 330 μm, scan time: 123 s) with varying flip angles (FA, 5°, 15°, 30°, 50°, and 70°) pre-contrast and FA of 15° post-contrast for a period of 25–35 min (all other parameters are as stated above). At the same day, while still anesthetized ovaries were also imaged using the MVX10 stereomicroscope (Olympus, Tokyo, Japan). Mice were injected with hCG and then allowed to recover from anesthesia by injection of Antisedan (atipamezole hydrochloride, 1 mg/kg, Zoetis, New Jersey, USA). On the next day, upon ovulation, mice were anesthetized by Isoflurane, injected with the fluorescent marker FITC-Dextran (500,000 KD, Sigma-Aldrich, Rehovot, Israel) and imaged again in the stereomicroscope (Figure 1C). Based on the microscopy images, follicles that transformed into CL were identified, and regions of interest were selected for further MRI image analysis.

### Data Analysis

Voxel-by-voxel data analysis was performed using purpose-written MATLAB (Math Works Inc. Massachusetts, USA) scripts to generate R_1_ maps and subsequently concentration maps of b-BSA-GdDTPA in the ovary region of interest as described (41). Briefly, pre-contrast R_1_ maps were derived from the variable FA data by a non-linear fit to the following equation:

(1) $$I\left(a\right)=\;\frac{M_{0}sina(1-e^{-TR•R_{1pre}})}{1-\cos a•e^{-TR•R_{1pre}}}$$

Where *I* is the signal intensity as a function of the pulse FA α, and the pre-exponent term, M_0_, includes contributions from both the spin density and the T_2_ relaxation.

Post-contrast *R*_1_ values (*R*_1post_) were calculated from pre- and post-contrast 3D-GE signal intensities:

(2) $$\frac{I_{\left(pre\right)}}{I_{\left(post\right)}}=\frac{M_{0}\sin a(\frac{1-e^{-TR•R_{1(pre)}}}{1-\cos a•e^{-TR•R_{1(pre)}}})}{M_{0}\sin a(\frac{1-e^{-TR•R_{1(post)}}}{1-\cos a•e^{-TR•R_{1(post)}}})}$$

Concentrations were calculated based on the relaxivity (*r*_1_) of biotin-BSA-GdDTPA (that was measured for each batch separately) with the following equation:

(3) $$c\left(t\right)=\;r1^{-1}(R_{1post}-R_{1pre})$$

Two vascular properties were derived by linear regression from the dynamic change in concentration during the first 15 min post-contrast: fractional blood volume (fBV, also referred to as blood volume), calculated as the ratio between the extrapolated concentration of contrast agent at the time of administration and the initial concentration in the blood, and permeability surface area product (PS, also referred to as permeability; units: min^−1^), calculated as the initial rate of contrast accumulation normalized to initial blood concentration (Supplementary Figure 1) (41). After fBV and PS maps were calculated for the ovary ROI, histograms were generated for fBV (range: 0–0.1 or 0.5) and PS (range: 0–0.02 min^−1^) values and plotted in 50 bins. Histograms are presented in voxel numbers per bin and in percentage of voxels per bin formats.

For the calculation of late enhancement and late clearance, the rate of changes in concentration of b-BSA-GdDTPA over the 25–35 min post-contrast was derived by linear regression. To remove spikes of overestimated contrast concentration, a maximum threshold of 10 mM was set (<5% of the ROI voxels, distribution of these voxels is described in Supplementary Figure 2). This threshold was set for observation purposes, i.e., to facilitate identification of follicles.

### Immunofluorescence and Immunohistochemistry

After MRI ovaries were collected, fixed in 4% paraformaldehyde, and embedded in paraffin blocks, 4-μm sections were made. Ovaries that were imaged through the MRI-compatible imaging window were taken at the same orientation as they were placed in the window, and 4-μm sections were made from the part of the ovary that was imaged by the stereomicroscope, to match the fluorescent intravital images. Hematoxylin and eosin (H&E) staining was done for one slide from each ovary.

Immunofluorescence was used for validation of blood volume and permeability. Intravascular and extravasated b-BSA-GdDTPA was visualized using Streptavidin Cy2 (Jackson Immunoreserch, PA, USA), whereas intravascular BSA-ROX could be observed directly in paraffin sections.

### Statistical Analysis

For the experiments of 36–48 h post PMSG vs. control mice, a linear mixed effects model with treatment as a fixed factor, and mouse and ovary as random factors, was used to calculate the statistical significance difference in averaged fBV and PS between the groups. The statistical significance of histograms was calculated for each bin separately with unpaired Student's *t*-test (*p* < 0.05). In cases where two ovaries from the same animal were imaged, they were averaged.

For the DF selection vs. CL formation experiments, a linear mixed effects model with treatment as a fixed factor, and mouse and ovary as random factors, was used to calculate the statistical significance difference in averaged fBV and PS between the two time points for the same subjects. Again, the statistical significance of histograms was calculated for each bin separately with a paired Student's *t*-test.

For DF vs. SF experiments, a paired Student's *t*-test was used to test the difference in averaged fBV and PS between the two sub-populations.

## Results

The angiogenic process that accompanies DF selection was studied against three relative process: ovarian angiogenesis in non-injected mice, angiogenesis of CL formation, and angiogenesis of SF (Supplementary Figure 2).

### DF Angiogenesis Shows Low Blood Volume and Low Permeability

Blood volume and permeability were derived from the dynamic changes in tissue concentration of b-BSA-GdDTPA, while marking a region of interest around the ovary. Ovarian average blood volume and average permeability were calculated according to the average signal intensity in the ovary. These values were then compared between ovaries of sexually immature mice that were imaged at 36–48 h after PMSG injection, the time of DF selection, and ovaries of same-age non-injected mice, in which DF selection does not take place (Figure 1A; a representative time lapse movie of post-contrast injection T_1_-weighted images, describing signal enhancement over time is shown in Supplemental Digital Content 1). The average blood volume and permeability at the time of DF selection were not statistically different (Figure 1B). Voxel-by-voxel maps of blood volume and permeability showed that ovaries that contained DF showed more voxels with high blood volume values and less voxels with high permeability values (Figure 1C). Histogram representation of the voxel-by-voxel maps showed an increase in the number of voxels at the low levels of blood volume and permeability after DF selection. However, no changes were detected upon normalization of the histograms to the size of the tissue (Figure 1D). The visualization of b-BSA-GdDTPA in histological sections of ovaries that were previously imaged by the MRI, using a fluorescently conjugated streptavidin, showed an accumulation of the contrast agent in the antrum of large follicles in PMSG-injected mice as well as in large follicles of non-injected controls. This was evident in the percent area of the ovary that was stained for b-BSA-GdDTPA, which was similar in the two groups (Figure 1E; separate channels of this staining and higher-magnification images are shown in Supplementary Figure 3).

### CL Demonstrate Increased Blood Volume but Similar Permeability Compared to DF

**Figure 2:**
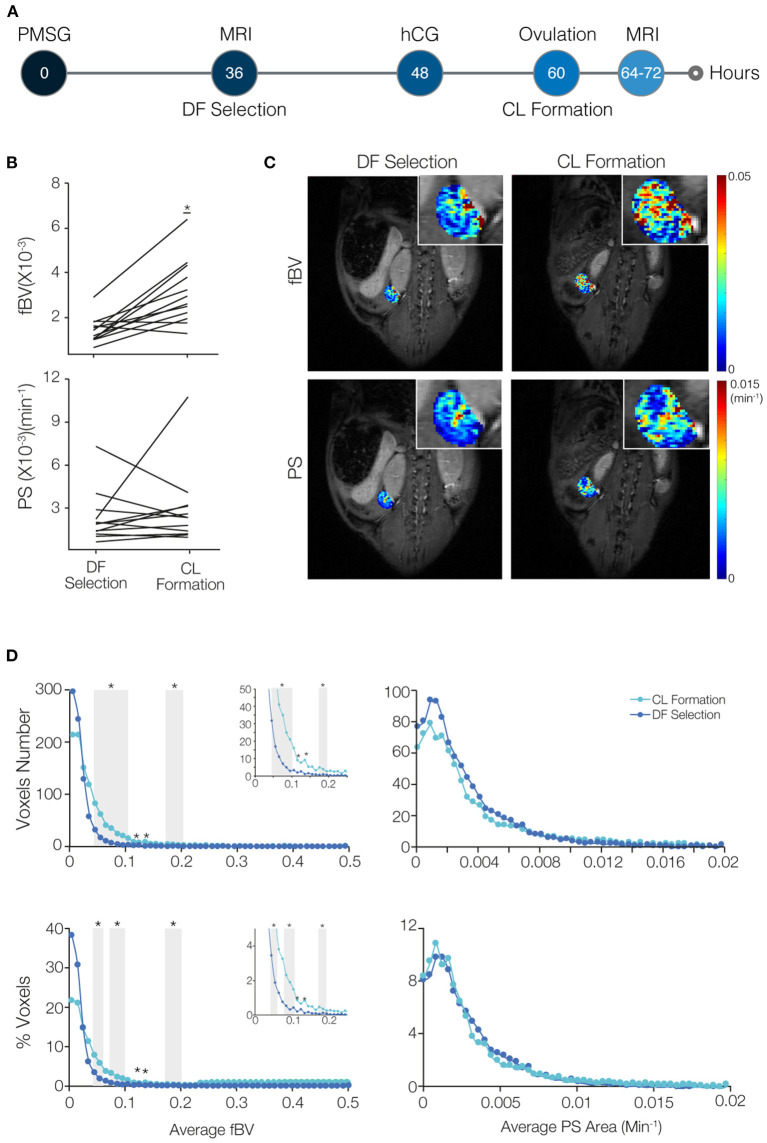
Blood volume increases while permeability surface area remains restrained in the transition of the dominant follicle (DF) to form the corpus luteum (CL). **(A)** Mice were imaged by DCE-MRI soon after DF selection (36–48 h after PMSG injection) and then injected with hCG. On the next day, after ovulation occurred and at the time of CL formation, mice were imaged again (*n* = 8 mice, 12 ovaries). **(B)** Fractional blood volume (fBV) and permeability surface area product (PS) averaged over the entire ovary voxels. Significant change is indicated (* indicates p-value = 0.0006). **(C)** Maps of fBV and PS with enlarged ovary region in the inset. **(D)** Histogram analysis of the total number and percent of voxels in each value range of fBV and PS. Ranges with significant increase in voxels with low values of fBV are indicated by gray background (* indicates *p*-value < 0.05). Inserts show the enlargement of the histogram at the fBV range of 0–0.25.

To compare blood volume and permeability in the transition from DF to CL, we imaged the same mouse at two time points, first at the time of DF selection (36–48 h after PMSG injection) and later at the time of CL formation (about 5–13 h after ovulation, which is 17–25 h after hCG administration; Figure 2A). A significant elevation in ovarian average blood volume was seen in the CL, which matches the massive angiogenic process taking place during its formation (Figure 2B). However, no significant change in average permeability was observed. Maps of the vascular parameters also showed an increase in blood volume in several areas in the ovary but no change in permeability (Figure 2C). The histograms showed an increase in the number of voxels in low values of blood volume, and no change in permeability (Figure 2D). Immunofluorescent staining of the same ovaries validated these results, showing that blood vessels penetrating the forming CL had minimal permeability, while, in the same animal, the antrum of an antral follicles that did not ovulate had high accumulation of b-BSA-GdDTPA (Supplementary Figure 5).

### DF Present Low permeability and Late Enhancement Compared to SF

**Figure 3:**
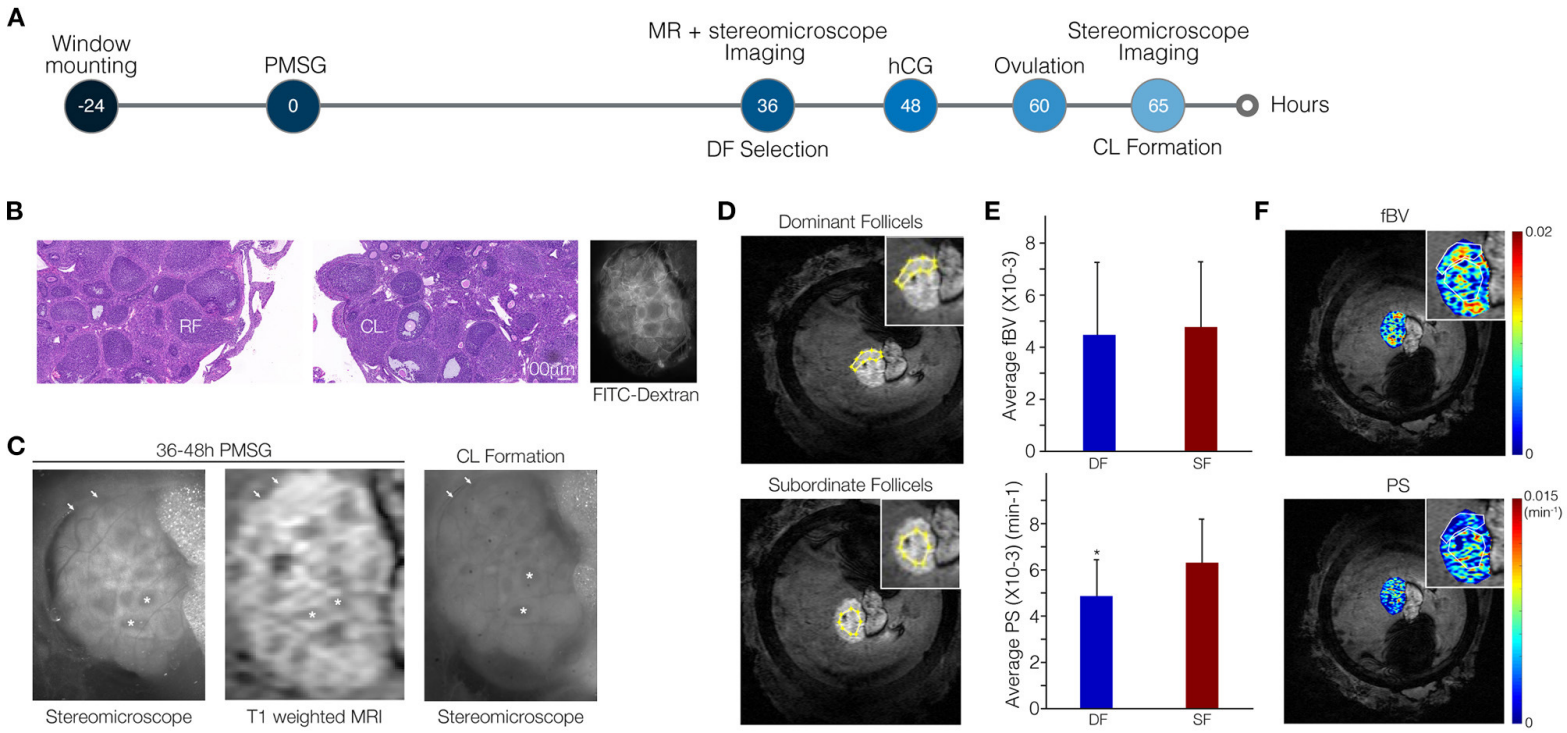
Vascular permeability is suppressed in dominant follicles (DFs) relative to subordinate follicles (SFs). **(A)** Ovarian follicles were imaged through the MRI-compatible ovarian imaging window at 36–48 h after PMSG injection by both DCE-MRI and fluorescent stereomicroscopy. Imaging was followed by hCG administration. On the following day, high-molecular-weight FITC-dextran was injected to the tail vein to visualize blood vessels and ovaries were imaged again under the stereomicroscope. **(B)** H&E stain showing the presence of ruptured follicles (RFs) and corpora lutea (CL) and FITC-dextran stereomicroscopy visualizes intact and functional blood vessels. **(C)** Fluorescent images and DCE-MRI (shown here at 6.5 min post-contrast). Follicles that lost their antrum (DF) to became CL are indicated by arrows. Follicles that did not ovulate and maintained their antrum (SF) are indicated by asterisks. **(D,E)** Representative example for regions of interest in the MRI images selected according to the fluorescent images **(D)** to analyze PS and fBV of DF and SF separately (**E**; *n* = 5, *p* = 0.02). **(F)** Corresponding pixel-by-pixel maps.

To increase resolution and enable distinction between blood vessel parameters of the DF and of SF, we made use of an MRI-compatible ovarian imaging window for both DCE-MRI and intra-vital fluorescent imaging (Figure 3A; a representative time lapse movie of post-injection T_1_ weighted images, describing signal enhancement over time is shown in Supplementary Digital Content 2).

We imaged the mice at the time of DF selection through the ovarian imaging window by high-resolution MRI. A stereomicroscope was employed to image these same ovaries again, at the following day, upon hCG-induced ovulation (Figure 3A). Viability of the ovary was confirmed by the presence of ruptured follicles and CL indicating that ovulation did take place in the imaged ovaries. Additionally, functionality of blood vessels in the window-mounted ovary was maintained as evident by spreading of the intravenously injected fluorophore in the FITC-dextran images (Figure 3B). Fluorescent and MRI images were comparable, allowing identification of follicles that transformed into CL and lost their antrum upon ovulation. These follicles were defined retrospectively as DF; follicles that did not lose their antrum were defined as SF (Figure 3C). The MRI resolution was improved dramatically from a voxel size of 117 × 117 × 500 μm to 39 × 39 × 330 μm, allowing the clear identification and analysis of single follicles. Regions including clusters of DF or SF were marked in the MRI images and blood volume and permeability were calculated (Figure 3D). We found that DF vessels exhibited significantly lower permeability as compared to SF. However, no significant change in blood volume was detected (Figure 3E). Lower permeability in the region of SF relative to DF region was also depicted in the vascular parameter maps (Figure 3F). Moreover, DF and SF, discriminated by their high or low LH receptor staining, respectively, showed similar CD34 but also similar alpha smooth muscle actin staining (Supplementary Figure 6).

**Figure 4:**
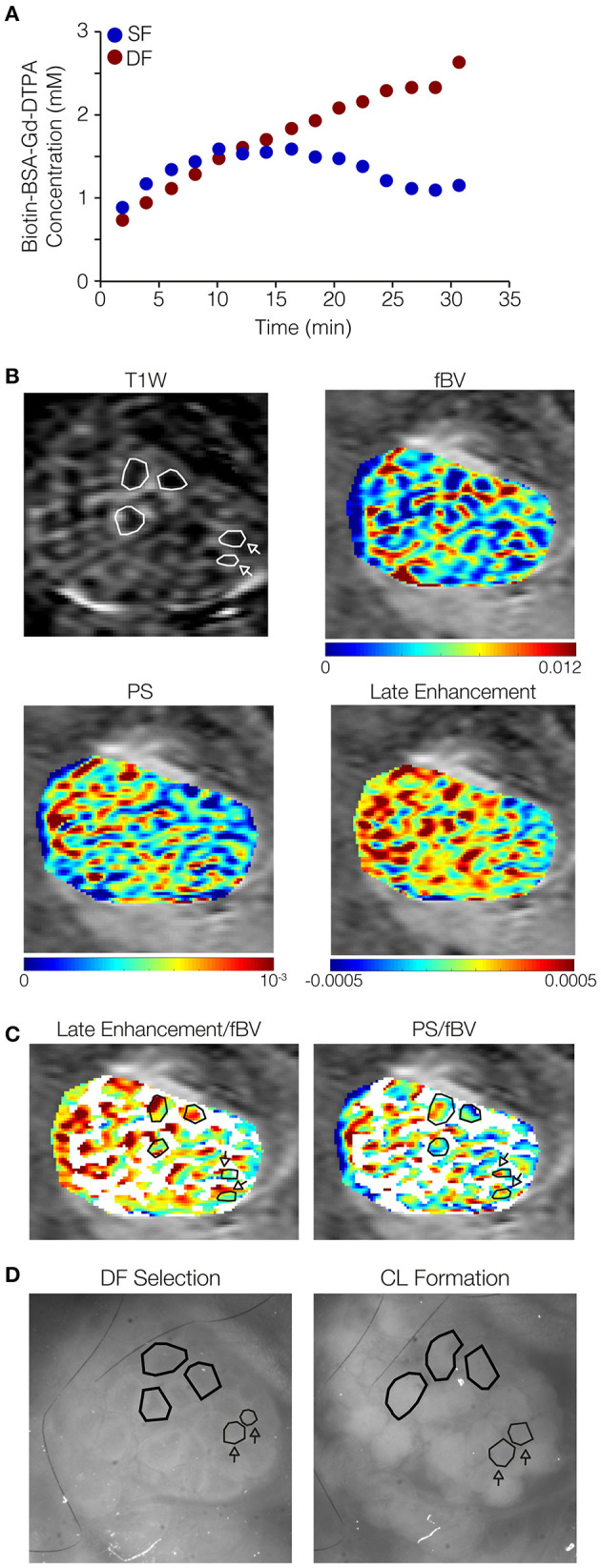
Dominant follicles (DFs) show late signal accumulation in their antrum while subordinate follicles (SFs) do not. Ovarian follicles were imaged through the ovarian imaging window after DF selection (36–48 h after PMSG injection), by MRI and fluorescent stereomicroscope, and again by stereomicroscope after formation of corpora lutea (CL; ~16 h after hCG administration). **(A)** Representative curves derived from a single mouse (out of five mice analyzed), of concentrations of b-BSA-GdDTPA over time for DF (red, continuous accumulation) and SF (blue, reaching a steady state followed by clearance). **(B)** Representative ovary imaged by DCE-MRI (3 min post-contrast image is shown); DF in polygons, SF in polygons indicated by arrows. Contrast accumulation follow-up for 35 min was used for the derivation of vascular parameters maps: fBV (initial contrast concentration), PS (accumulation rate at the first 15 min), and late enhancement (accumulation rate over that 25–35 min post-contrast). Color-coded late enhancement map shows positive enhancement (yellow through red) and zero to negative enhancement (stasis or clearance of contrast agent; green through blue). **(C)** PS and late enhancement maps overlayed with binary fBV map representing the highly vascularized layer around big follicles (white; selectively showing fBV values of 0.006 and above). Follicles indicated by polygons: positive late enhancement and low PS, identified by corresponding stereomicroscope images as follicles that lost their antrum and became CL. Follicles indicated by polygons indicated by arrows: zero or negative late enhancement and high PS, identified as follicles that did not lose their antrum upon ovulation. **(D)** Stereomicroscope images (without a fluorescent marker) of the same ovary at DF selection and CL formation.

The vascular parameters, blood volume (fBV) and permeability (PS), were derived from the early dynamics (15 min) of b-BSA-GdDTPA. Further analysis of the later dynamics revealed that b-BSA-GdDTPA continued its accumulation in DF throughout the time of the experiment (up to 33 min post-contrast), while the concentration of b-BSA-GdDTPA in SF reached a steady state 6–16 min after injection and then showed a slow clearance (Figure 4A). Color-coded maps of early (PS) and late contrast accumulation were compared to fluorescent images taken before and after ovulation (Figures 4B–D). This comparison depicted late enhancing ovarian foci with low permeability that correlated with DF. Other areas showed a decrease or no change in contrast agent concentration and correlated to SF (Figure 4C).

A summary of the findings in this study can be found in Table 1.

**Table 1:** Results summary of fBV and PS in different experiments.

	**fBV**	**PS**	**Delayed Enhancement**	**Figure Presenting Results**
Ovaries of non-injected mice compared to ovaries of PMSG-injected mice	↑	↑		1
Ovaries of PMSG-injected mice compared to ovaries of PMSG +hCG mouse	↓	=		2
DF vs. SF	=	↓	↑	3, 4

*A change in a value is indicated by an arrow (increase or decrease). All changes are in respect to the ovary of the PMSG injected mouse. No change is indicated by (=)*.

## Discussion

In this study, we focused on functional vascular changes that characterize early stages of DF selection. We utilized DCE-MRI for the three-dimensional non-invasive visualization of the mouse ovarian vasculature combined with high-resolution imaging performed using an MRI-compatible ovarian window reported by us previously recently (40, 43, 44). The window spatially holds the ovary to reduce motion and allows a smaller field of view. Combined with a matched surface coil for detection, it improves the signal-to-noise ratio. This multi-imaging modality enabled evaluation of blood vessel properties and observation of dynamic patterns of contrast agent distribution at the follicle level, allowing comparison between different populations of follicles in a way that was not possible in previously published MRI experiments in murine ovaries (33, 35–39).

Increase in blood volume is a hallmark of the DF (25, 45). In concordance, the increase in blood volume was clearly visible when looking at the voxel-by-voxel vascular parameter maps of ovaries of PMSG vs. non-injected animals. The histogram analysis revealed that the angiogenic process at this stage was characterized by elevated low values of blood volume, suggesting an increase in small vessels or a minor increase in vessels width. However, normalization of the histograms to the tissue size showed that the growth in vessels was proportional to the growth in tissue volume. This could be explained by the restriction of the vasculature to the surface of the follicles at this stage and is in concordance with the lack of statistical significance in average ovarian blood volume.

Upon CL formation, we detected a substantial increase in blood volume, as expected. In contrast to the vessels of the DF, the vessels of the forming CL were characterized by higher values of blood volume, indicating the formation of either more vessels or larger vessels in this process. In this case, their growth was not proportional but higher than the growth in tissue size. In previous studies done in our lab, arterial spin labeling MRI was used to assess ovarian perfusion at a time window of up to 10 h after hCG administration (33, 34). Perfusion was shown to be decreased at 2 h after hCG injection, followed by a transient rise that was proportional to the growth in tissue weight. However, these experiments were done at an earlier time point prior to ovulation.

The moderate permeability seen in the angiogenesis associated with DF selection and CL formation might point at a quick stabilization of the forming vessels. This could not be explained by a change in αSMA, but could be attributed to other molecular factors. Fast stabilization of the DF and the CL vasculature could have a functional advantage in allowing higher efficiency of hormone transmission. Small molecules and proteins up to 100 kDa are transported in and out the follicle by osmosis (46, 47). Our MRI contrast agent, b-BSA-GdDTPA, being about 82 kDa in size, represents such proteins. One example for a small protein produced by the follicle in association to dominance establishment is inhibin. Inhibin (31 kDa) is a gonadal origin hormone that negatively feeds back the synthesis and secretion of the pituitary FSH (48). Since one of the major characteristic properties of the DF is its ability to shift its dependence from FSH to LH while the SFs are still dependent on FSH for their survival, inhibin is a key factor in DF selection. The low permeability of the DF may contribute to the efficient transmission of inhibin from the follicle to the peripheral blood system, thus contributing to the declining levels of FSH.

It was previously shown that in the CL, LH-induced VEGFA upregulation directly influences an increase in permeability by downregulating VE-cadherin, occluding, and claudin5 (49), allegedly in contrast to our findings. However, these experiments were done at a later stage of CL developments (after oocyte fertilization, at the early stages of pregnancy) whereas our experiments were conducted as soon as 5 h after ovulation. It was also shown that blood vessels of the CL have increased permeability as soon as 2–10 h after hCG injection (50–52). CL permeability was also shown to facilitate the infiltration of immune cells, which have a role in ovulation, CL function, and angiogenesis (53–55). Therefore, our results may suggest a permeability switch occurring in the CL, starting with high permeability, which allows the infiltration of immune cells, followed by low permeability. An additional increase in permeability may be induced in early stages of pregnancy. It is also important to note that the results of our study do not suggest that vessels are not permeable during the early stage of CL formation, but that the permeability that accompanies CL formation is similar to permeability during DF selection, which, in both cases, is kept moderate.

We further detected a differential pattern of contrast agent accumulation between DF and SF during DCE-MRI. Accumulation of b-BSA-GdDTPA in DF was continuous (up to 33 min after injection of b-BSA-GdDTPA), while in SF, clearance was observed at around 25 min. In addition to the decrease in permeability that was detected in DF, this result could point at a difference in extracellular matrix (ECM) components of the DF, leading to a slower passage from the blood vessels to the antrum. An ECM component that is known to be expressed differentially between DF and SF is fibronectin, which increases in the theca cell layer and stroma with follicular development while its level in the granulosa cells decreases, making the follicle more rigid and less permissive for large solutes to travel from the blood vessels to the antrum (56). Nevertheless, the involvement of fibronectin or any other ECM component in the late enhancement phenomena and low permeability of the DF is to be elucidated.

Our results suggest measurable vasculature functional parameters that discriminate DF from SF soon after their selection. This discrimination may be clinically relevant in cases of poor responders to hormonal stimulation during assisted reproductive technologies. It was reported that a number of 15 retrieved oocytes during *in vitro* fertilization procedures is optimal for live birth outcome (57). However, in some cases, patients do not reach this number and are defined as poor ovarian responders with a low prognosis (58, 59). The method described in this study can assist in unveiling the molecular mechanisms and physiological processes that occur in low prognosis patients with the intention to resolve them. In the clinics, DFs are usually identified by size deviation from the SF using ultrasound. The functional vascular parameters evaluated here by MRI may offer DF detection prior to size deviation. Furthermore, discrimination between DF and SF and the ability to detect DF at an early developmental stage in the mouse ovary opens the possibility for investigation of dominance establishment following different intervention as well as in transgenic mouse models.

To conclude, in this study, we used *in vivo* multi-modality imaging of the vasculature to track the dynamics of blood flow identifying moderate permeability and late enhancement as hallmarks of the DF. We further suggested the employment of these properties as predictive markers for DF as early as at the initial stage of their selection.

## Data Availability Statement

The raw data supporting the conclusions of this article will be made available by the authors, without undue reservation.

## Ethics Statement

The animal study was reviewed and approved by Weizmann Institute IACUC.

## Author Contributions

LF-A conducted all experiments and data analysis. IB guided and assisted in MRI experiments. HD wrote MATLAB codes for data analysis and assisted with data analysis. FB designed the ovarian imaging window and assisted in surgery of animals. RR performed statistical analysis. MN and ND supervised the work. All authors contributed to the article and approved the submitted version.

## Conflict of Interest

The authors declare that the research was conducted in the absence of any commercial or financial relationships that could be construed as a potential conflict of interest.

## Publisher's Note

All claims expressed in this article are solely those of the authors and do not necessarily represent those of their affiliated organizations, or those of the publisher, the editors and the reviewers. Any product that may be evaluated in this article, or claim that may be made by its manufacturer, is not guaranteed or endorsed by the publisher.
