# Supplementary material for: Prediction of Ovarian Follicular Dominance by MRI Phenotyping of Hormonally Induced Vascular Remodeling
Source: Front Med (Lausanne). 2021 Aug 20;8:711810. doi: 10.3389/fmed.2021.711810 (PMC8417579; doi:10.3389/fmed.2021.711810)
Supplement: Supplementary file 1 [file Data_Sheet_1.pdf]

Supplementary Material

1. Supplementary Methods

Immunohistochemistry

Co-staining of LH receptor (LHR) and CD34 or alpha Smooth muscle actin (αSMA) was done on parallel FFPE sections of ovaries that were imaged in the MRI 36-48 hours after PMSG injection. Slides were de-paraffinized and antigen retrieval was done using Tris-EDTA (PH=9) for LHR, and citric acid (PH=6) for CD34 and αSMA. Blocking for unspecific binding was done with 20% Normal Horse Serum (NHS), 0.02% TX-100 in PBS. Primary antibody (LHR 1:150 Alomone, Jerusalem, Israel, ALR-010; CD34 1:100 Cederlane, Ontario, Canada, CL8927AP; αSMA 1:500, GeneTex, California, USA GTX100034) in 2% NHS and 0.02% TX-100 were incubated overnight. Slides were then incubated with matching secondary antibody and counterstained with Hoechst (Invitrogen, Waltham, Massachusetts, USA).

2. Supplementary Figures and Digital Content

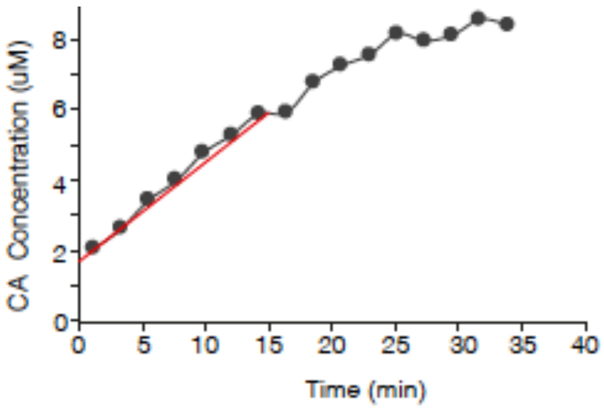

Figure S1. Represetative contrast accumulation curve showing the dynamic changes of the MRI contrast agent, biotin-BSA-Gd-DTPA, in the mouse ovary over time. Linear regression

of the first 15 min post contrast (red) was used for calculation of two vascular parameters, fractional blood volume (fBV; concentration at time zero, namely the intercept, divided by initial concentration in the blood) and permeability surface area product (PS, the change rate, namely the slope, divided by initial concentration in the blood). Similar processing was used to generate fBV and PS maps.

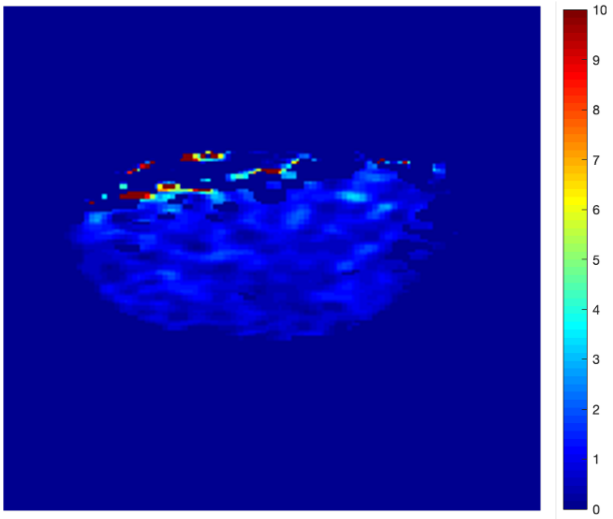

Figure S2. A pixel by pixel map describing calculated contrast agent concentration. Pixels with above 10mM concentration are depicted in red, essentially out of the ovary.

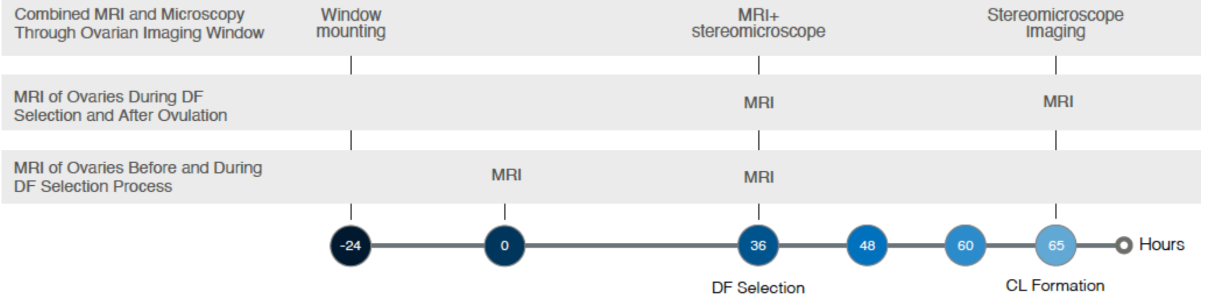

Figure S3: A schematic illustration of experimental setups.

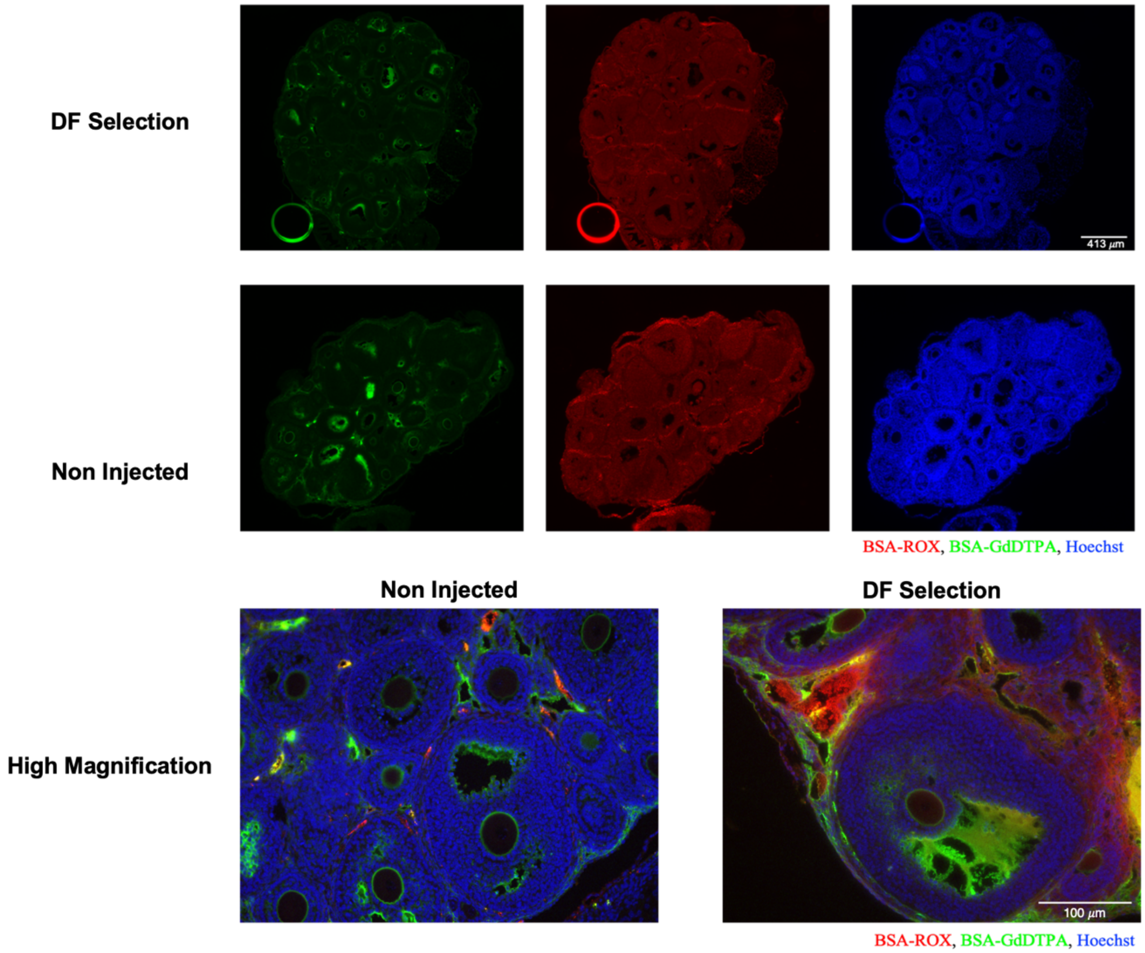

Figure S4. Immunofluorescent staining of b-BSA-GdDTPA (intra and extravascular at 35 minutes after injection; visualized using fluorescent streptavidin, green) and for BSA-ROX (confined mainly to the blood vessels at 2 minutes after injection, red) in ovaries of mice that were imaged by MRI 36-48 hours after PMSG injection and non-injected mice.

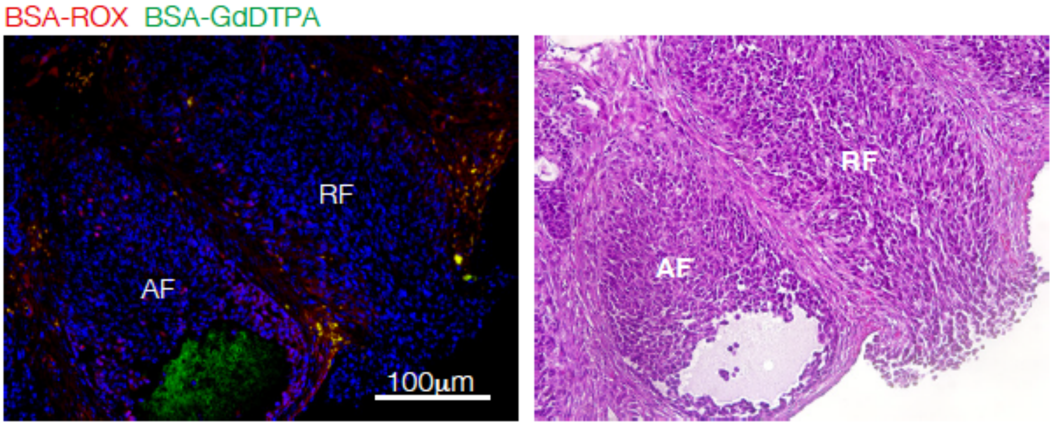

Figure S5. Immunofluorescent staining of b-BSA-GdDTPA (intra and extravascular at 35 minutes after injection; visualized using fluorescent streptavidin, green) and for BSA-ROX (confined mainly to the blood vessels at 2 minutes after injection, red) in ovaries of mice that were imaged by MRI at the time of CL formation showing minimal permeability of growing vessels in ruptured follicle (RF) and high permeability and accumulation of the contrast agent in the antrum of antral follicle (AF). Matching H&E staining shows the ruptured follicle and antral follicle.

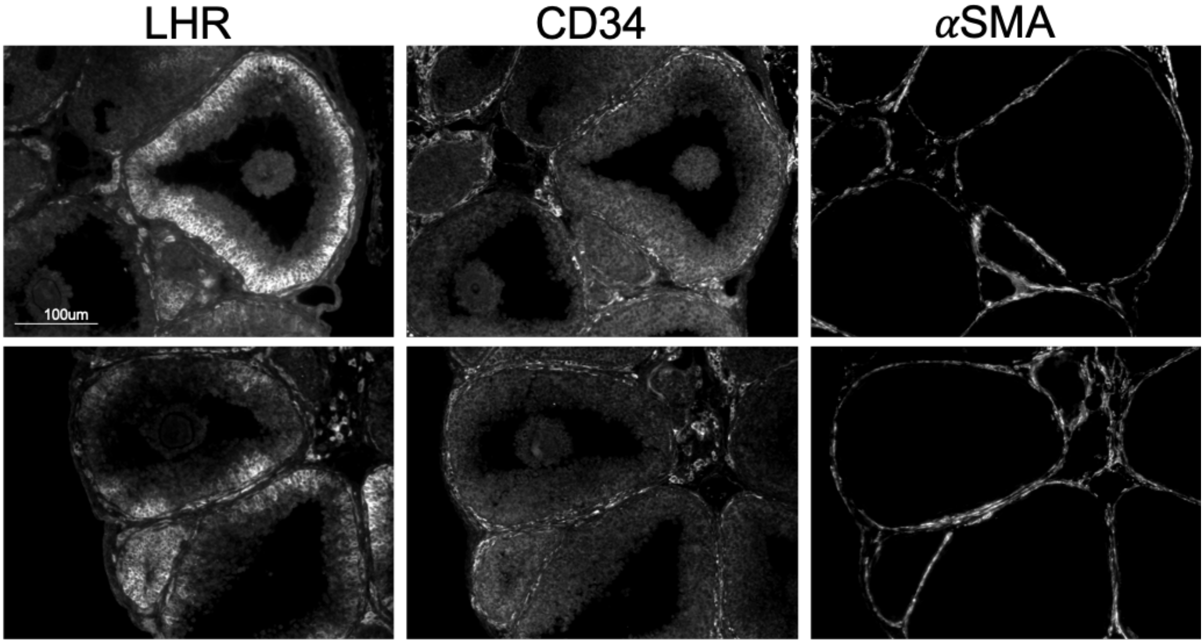

Figure S6: Parallel section of ovaries that were collected from mice 48 hours PMSG injection were stained for CD34, αSMA and LHR to evaluate the difference in newly formed blood vessels and in the vascular wall stability in DF and SF as distinguished by the LHR staining. No significant difference between CD34 or αSMA expression was found between DF and SF. representative example of DF in the upper panel and SF in the lower panel.

Supplementary Digital Content 1: A representative time lapse movie of post contrast injection T_1 weighted images, describing signal enhancement over time.

Supplementary Digital Content 2: A representative time lapse movie of post injection T_1 weighted images, describing signal enhancement over time
